# 2350. LiaF is required for LiaX mediated protection against Daptomycin and LL-37 in Enterococcus faecalis OG1RF

**DOI:** 10.1093/ofid/ofac492.157

**Published:** 2022-12-15

**Authors:** Diana Panesso Botero, Kavindra Singh, Sara-Isabel Gomez-Villegas, Ayesha Khan, Adeline Supande, April Nguyen, Sandra Rincon, Kara S Hood, An Dinh, Alexander Deyanov, William R Miller, Yousif Shamoo, Cecilia Tran, Cesar A Arias

**Affiliations:** Houston Methodist Research Institute, Houston, Texas; Houston methodist research institute, Houston, Texas; Houston methodist research institute, Houston, Texas; Clinica Alemana, santiago de chile, Region Metropolitana, Chile; Rice university, Houston, Texas; University of Texas Health Science Center, Houston, Texas; Universidad El Bosque, Bogota, Distrito Capital de Bogota, Colombia; Houston methodist research institute, Houston, Texas; Houston methodist research institute, Houston, Texas; Houston methodist research institute, Houston, Texas; Houston Methodist Hospital and Weill Cornell Medical College, Houston, Texas; Rice university, Houston, Texas; Houston Methodist Hospital, Houston, TX; Houston Methodist Hospital and Weill Cornell Medical College, Houston, Texas

## Abstract

**Background:**

Daptomycin (DAP) resistance in enterococci is regulated by the LiaFSR three-component regulatory system. Mutations in *liaF* have been linked to activation of this system, with increased expression of the protein LiaX, and resistance to DAP in clinical isolates. LiaX functions by recognizing DAP in the extracellular medium, and serves as a signal transduction molecule. However, the role of LiaF in signaling and its relationship with LiaX are unknown.

**Methods:**

We generated a *liaF* null mutant in *E. faecalis* OG117 by adding four stop codons at amino acid positions 11–14 (OG117liaF*_11-14_) using a CRISPR-Cas9 system. The mutant was complemented by restoring wild-type *liaF* in its chromosomal location (OG117liaF*_11-14_::*liaF*). Mutants were confirmed with whole genome sequencing (WGS). Anionic phospholipid (AP) microdomain distribution was assessed on microscopy using 10-*N*-nonyl-acridine-orange (NAO). LiaFSR activation was assessed by surface expression of LiaX via ELISA. DAP MICs were performed by broth microdilution in the presence/absence of exogenous LiaX (eLiaX). LL37 (50µg/ml) and DAP (1.5µg/ml) killing assays with eLiaX were also performed in triplicate.

**Results:**

Truncation of *liaF* did not have any effect on DAP MICs, AP microdomain distribution or LiaX surface expression compared to OG117. No additional mutations were observed by WGS in the mutants. In the presence eLiaX, DAP MIC increased 8-fold in wild-type OG117 but remained the same (1 µg/mL) in OG117liaF*_11-14_. Complementation of *liaF* restored the increase in DAP MIC in the presence of eLiaX. In the LL37 killing assay, survival of OG117 was significantly increased on the addition of eLiaX (2.9% vs 11.8%, respectively, *P* < .002). Addition of eLiaX was unable to rescue OG117liaF*_11-14_ in the presence of LL-37 (2.4% vs 2.5% respectively, *P*=0.51), but was able to increase survival in the complemented strain (2.8% vs 7.6%, respectively, *P* < .002). A similar trend was seen in the DAP killing assay, with increased survival for OG117 (8.7% vs 20%) and OG117liaF*_11-14_::*liaF* (11.6% vs 17%), but not OG117liaF*_11-14_ (0.38% vs 1%).

**Conclusion:**

LiaF is required for full tolerance to the cathelicidin LL37 and DAP in *E. faecalis* OG1RF. These results suggest that LiaF may interact with LiaX as a part of the LiaFSR stress response.

**Disclosures:**

**William R. Miller, MD**, Entasis: Grant/Research Support|UpToDate: Honoraria **Cesar A. Arias, MD, PhD**, Entasis: Grant/Research Support|MeMed Diagnostics Ltd: Grant/Research Support|Merck: Grant/Research Support.

